# The unique and synergistic effects of social isolation and loneliness on 20-years mortality risks in older men and women

**DOI:** 10.3389/fpubh.2024.1432701

**Published:** 2024-10-16

**Authors:** Marja Aartsen, Hanna Vangen, George Pavlidis, Thomas Hansen, Iuliana Precupetu

**Affiliations:** ^1^NOVA – Norwegian Social Research, Oslo Metropolitan University, Oslo, Norway; ^2^Institution of Social and Psychological Studies, Karlstad University, Karlstad, Sweden; ^3^Department of Mental Health and Suicide, Norwegian Institute of Public Health, Oslo, Norway; ^4^Research Institute for Quality of Life, Bucharest, Romania; ^5^Research Institute of the University of Bucharest, Bucharest, Romania

**Keywords:** loneliness, social isolation, mortality, older adults, aging, longitudinal study, Norway

## Abstract

**Introduction:**

This study investigates the individual and combined impacts of loneliness and social isolation on 20-year mortality risks among older men and women.

**Methods:**

Utilizing data from the Norwegian Life Course, Ageing, and Generation study (NorLAG) carried out in 2002, 2007 and 2017, we assessed loneliness via direct and indirect questions, and social isolation through factors like partnership status and contact frequency with family and friends. Yearly information on mortality was derived from the national registries and was available until November 2022. Gender-stratified Cox regression models adjusted for age and other risk factors were employed.

**Results:**

Of the 11,028 unique respondents, 9,952 participants were included in the study sample, 1,008 (19.8%) women and 1,295 (26.6%) men died. In the fully adjusted models including indirectly assessed loneliness, social isolation increased the 20-year mortality risk by 16% (HR = 1.16, 95% CI 1.09–1.24) for women and 15% (HR = 1.15, 95% CI 1.09–1.21) for men. This effect was primarily driven by the absence of a partner and little contact with children for both genders. Loneliness measured with indirect questions lost its significant association with mortality after adjusting for social isolation and other factors in both genders. However, for men, reporting loneliness via a direct question was associated with a higher mortality risk, even in the fully controlled models (HR = 1.20, 96% CI 1.06–1.36). Interactions between loneliness and social isolation were not, or only borderline significantly, associated with mortality risks in the fully controlled models.

**Discussion:**

Social isolation, but not loneliness measured with indirect questions are associated with a 15–16% higher mortality risk in both men and women. However, loneliness assessed with a direct question is associated with increased mortality in men, even after controlling for social isolation and other relevant factors, which might suggest that men may deny loneliness, unless it is (very) severe. These findings emphasize the importance of methodological precision in the measurement of loneliness and social isolation.

## Introduction

It is widely acknowledged that social connections are indispensable for physical, cognitive, and mental health [e.g., (1, 2)]. The COVID-19 pandemic has brought increased attention to research on loneliness and social isolation, pushing it even higher on the research agenda. Berkman et al. (3) developed a conceptual model that connects social relations to health outcomes through support, engagement, and resource access, while also influencing health behaviors. Matud et al. (4) and Thoits (5) similarly recognize the critical role of support in social ties for health but go one step further by distinguishing between the actual support received versus the general sense of available support (perceived support) in the network of social relations. They argue that perceived support exerts the most powerful effects on health.

More recently, scholars started to link subjective and objective aspects of social relations to mortality, and concluded that both a perceived deficit of social relations (often referred to as loneliness) as well as an actual deficit of social relations (often referred to as social isolation) increases mortality (6, 7). However, substantial variations exist in the individual studies on mortality. For example, Tilvis et al. (8) find that loneliness, but not social isolation is associated with increased mortality, consistent with Thoits’ argument that perceived aspects of support are more important than actual support. Others find the opposite, that is, social isolation but not loneliness is associated with increased mortality (9–11). Yet others find that social isolation impacts mortality but only if mediated by loneliness (12). The precise mortality impact of social isolation and loneliness remains elusive, which may be due to a variety of reasons. The main aim of the present study is to better understand the unique and synergistic mortality risks of social isolation and loneliness, by considering potential reasons for inconsistent findings from mortality studies on loneliness and social isolation.

One inconsistency in mortality studies on social isolation and loneliness is the wide variety in assessments of social isolation and loneliness (13). Scholars generally agree that social isolation refers to an objective, quantifiable state in which a person has no, or only very few social relations. However, measurements of social isolation are often *ad-hoc* (14) and based on different heterogenous combinations of living arrangements (living alone or with partner), contacts in the wider social network (children, siblings, friends), and participation in society. Some studies combine these items into an index [e.g., (10, 11, 15)]. An index does not provide insight in the potential differential impact of the indicators on mortality. Iecovich et al. (16) therefore used disaggregated single variables (marital status, number of children and contact frequency, number of friends and contact frequency, and household size). Ward et al. (17) used the Berkman-Syme Social Network Index (18), an existing scale to assess social isolation, based on the number of social relations and the relative importance of the relations (e.g., intimate contacts received higher weights).

Loneliness on the other hand refers to the subjective negative feeling that occurs when there is a deficit in the social relations, either in the number or in the quality of social relations (19). Commonly used and validated measures for loneliness include single-item direct questions such as “Do you feel lonely” and scales consisting of indirect questions avoiding the word lonely such as in the UCLA loneliness scale (20) as well as in the De Jong Gierveld loneliness scale (21). While direct and indirect assessments of loneliness correlate highly (22), direct questions are also criticized as lonely people must openly admit that they are lonely, which is a taboo in many cultures. Therefore, it is unclear whether scores derived from different measures of loneliness reflect similar facets of loneliness or different degrees of severity with similar impacts on mortality.

Another reason for inconsistent findings may be that social isolation and loneliness are often investigated separately without taking potential synergy into account, even if both are included in the same study. Since loneliness and social isolation are correlated, ignoring potential synergies between the two constructs in the analytical models may lead to different outcomes, loss of predictive accuracy, and biased conclusions (23, 24). The few studies that consider synergies find that social isolation and loneliness reinforce each other’s effect on mortality (15, 17, 25). Finally, the effect of predictors on mortality tends to fade out over time, such that the longer ago the predictor was measured the weaker its association with mortality (26). Especially studies with longer follow-up may therefore fail to find significant effects if only baseline predictors are included. One study that includes time-varying values of loneliness and (indicators of) social isolation finds that household size, but not loneliness, is associated with mortality in men, whereas both loneliness and indicators of social isolation are unrelated to mortality in women (16).

In this study, we aim to further unravel the unique and synergistic impact of social isolation and loneliness on mortality by using a large sample of older people with long follow-up, use different measures of loneliness and social isolation, and investigate their unique and synergistic effects on mortality while adjusting for age and other pertinent mortality risk factors. We stratify the analyses by gender, to detect impacts that otherwise could remain undetected if opposite effects occur, as gender is associated with both the exposure variables (loneliness and social isolation) and the outcome variable, mortality (17, 27, 28).

## Materials and methods

### Data

Data are derived from the Norwegian Life Course, Ageing, and Generation study (NorLAG), a nation-wide population-based longitudinal survey carried out in 2002, 2007 and 2017 (29). Data are collected by means of computer assisted telephone interviews (CATI) supplemented with self-administered questionnaires and registry data. The survey data is combined with annual data from the public registers up to 2022. The total number of unique respondents in NorLAG is 11,028, and people are born between 1922 and 1966. The first wave included 5,555 people from 30 Norwegian municipalities aged 40–80 at the time of the interview. In the second wave, a refreshment sample was added to make the study representative for the older Norwegian population. Also younger birth cohorts (aged between 40 and 45) were included in addition to respondents from wave 1 (*N*_wave 2_ = 9,238). The third wave included respondents who participated either in wave 1 or wave 2 or both (*N*_wave 3_ = 6,099). The final study sample (*N* = 9,952, 51.0% were women) included all unique respondents with valid information for all relevant questions in at least one of the three waves. The 9.7% respondents that were excluded from our study because of non-response on relevant questions were less likely to be younger (OR = 0.98, 95% C.I. 0.97–0.98), female (OR = 0.91, 95% C.I. 0.83–0.98), having a high level of education (OR = 0.73, 95% C.I. 0.70–0.76), better mental (OR = 0.99, 95% C.I. 0.98–0.99) or physical health (OR = 0.98, 95% C.I. 0.98–0.99).

### Measures

Information on mortality was derived from the public registers, and in line with general data protection regulation. The year of death, but not the month or day was available for our analyses. Data on mortality was available for all NorLAG participants up to November 2022, independent on whether they participated in follow-up waves or not.

### Key predictors

*Loneliness* was assessed with a direct question including the word loneliness: “In the last week, I felt lonely” and with three indirect questions not including the word lonely, i.e., “There are many I can trust completely,” “I miss having a really close friend,” and “I find my circle of friends and acquaintances too limited.” Answers on the direct question were dichotomized into 1 (sometimes or always) and 0 (never or seldom). The three questions were derived from the validated De Jong Gierveld loneliness scale (21). Answering categories ranged from 1 (very much agree) to 5 (very much disagree) and were recoded such that a higher score was indicative of more loneliness. Answering “do not know” was also given the score 3 (more or less). The average score on the three questions was used in the analyses. Cronbach’s alpha for the loneliness scale was 0.64, 0.65 and 0.58 in wave 1, 2 and 3, respectively. Although this is rather low for a 3-item scale, we decided to use it as these were the only available in all three waves.

*Social isolation* was based on four items: having a partner yes (0) or no (1) and having no or less than monthly contact with each of the following categories: children, siblings, and friends. A score of 1 was provided for each category with whom people had less than monthly contact. The four items were used in the models separately and as an index ranging from 0 (at least monthly contact with children, siblings and friends and having a partner) to 4 (no or less than monthly contact with children, siblings, and friends during the last year and without partner).

### Control variables

*Age* was derived from the public registers and reflects the number of years since birth. *Income* is based on the individual income of the respondent after tax, which was derived from public registers and was available until November 2022. Because of the skewed distribution, income is recoded into deciles running from 1 (lowest 10%) to 10 (highest 10%). *Education* is measured on a five-point scale, ranging from 1 (primary education) to 5 (university and college education), which we for reasons of simplicity recoded into low (1 and 2), middle (3) and high (4 and 5). *Physical health* and *mental health* were assessed with a short form (SF-12) of the Health Survey (SF-36), a widely used and validated generic health measure (30). The SF-12 contains six items indicative of physical health and six items indicative of mental health. The physical health score includes items measuring physical functioning, role limitations due to physical health, bodily pain, and general health perceptions. The six items indicative of mental health include items measuring mental energy, social time, feeling peaceful and sad. In line with instructions (30), items were weighted and summed into the physical component scale (PCS-12) and a mental component scale (MCS-12). The two scale scores were standardized with a mean set to 50 and SD to 10. Higher scores indicate better physical or mental health.

### Handling of missing values

NorLAG includes complete mortality data derived from the annual registers (from 2002 until November 2022) for all participants. Since the survey data was only collected three times (2002, 2007 and 2017) we do not have yearly updated values on the survey variables between the waves. Also, register data on income is often missing in the year of death (deceased people do not pay tax). Missing data were handled by a simple and commonly used method of imputing the missing information for the years in between the waves by carrying forward the last survey observation of the independent variables until the next observation or until death, or until the end of study (in 2022) in case of survival. A robustness test was carried out including only the individuals who had at least one observation, but without carrying forward the last observation to the next round. All people who died within the first 12 months of participation were also excluded (*n* = 42) to ensure that findings are not influenced by reversed causation, for example by people who self-isolated and felt lonely because of their expected closeness to death.

### Analytical strategy

A series of gender stratified stepwise Cox regression models with time varying covariates was conducted for 5,079 women and 4,873 men. Age was included in all models. Model 1 (M1) estimates the main effect of the indirect loneliness questions on mortality and M2 the main effects of the four social isolation indicators. M3 combines M1 and M2 to estimate the unique effects loneliness and social isolation on mortality. M4 adds to M3 all other control variables (i.e., mental and physical health, education, and income). In M5, the four social isolation items are replaced by the social isolation index, and further includes loneliness and all control variables. Finally, in M6 we added the interaction between social isolation (index) and loneliness (mean of indirect questions) to evaluate whether an interaction between loneliness and social isolation would further increase mortality beyond their unique effects and the effects of all control variables. We repeated the same procedure for the direct loneliness question.

## Results

**Figure 1:**
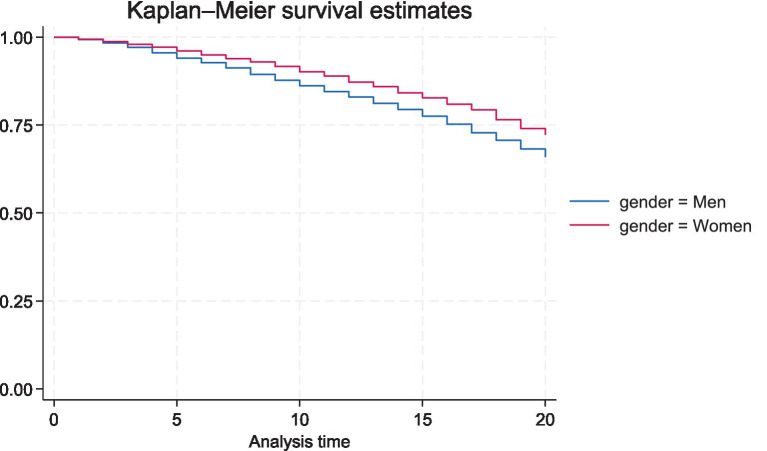
Kaplan–Meier survival curves for men and women.

Of the study sample, 1,008 (19.8%) women and 1,295 (26.6%) men died during follow-up, and 29.3% of the women and 22.5% of the men were classified as lonely based on the direct question (Table 1). Almost 80% of the men and women had at least monthly contact with their children, 46% had regular contact with siblings, while 91% of the women and 86% of the men had at least monthly contact with friends. Little less than one-third of the men and almost 40% of the women live without a partner. The average age at the start of the study was 56.9 years. The Kaplan–Meier survival curves (Figure 1) depict the probability of surviving in the 20 years of follow-up for men and women (no gender difference in age at baseline). There is a clear gender effect with men having greater likelihood to die during follow-up.

**Table 1:** Descriptive characteristics of the study sample (*N* = 9,953).

	N, %, M (SD)	Women	Men
Total number	9,952	5,079	4,873
Number of people deceased (%)	2,303 (23.1)	1,008 (19.8)	1,295 (26.6)
Loneliness			
Indirect questions, range 1–5 (M, SD)	1.9 (1.02)	1.9 (1.02)	2.0 (1.01)
Direct question, (% lonely)	26.0	29.3	22.5
Social isolation index	1.2 (0.95)	1.2 (0.93)	1.2 (0.97)
Children, at least monthly contact (%)	78.6	80.7	76.4
Siblings, at least monthly contact (%)	46.3	46.7	45.8
Friends, at least monthly contact (%)	88.5	90.5	86.4
No partner (%)	34.6	39.7	29.3
Age (M, SD)	56.9 (11.0)	56.5 (11.0)	57.3 (10.9)
Mental health, range 1–100 (M, SD)	54.9 (7.8)	54.3 (8.2)	55.6 (7.3)
Physical health, range 1–100 (M,SD)	48.0 (10.7)	46.8 (11.5)	49.2 (9.7)
Income after tax. Deciles 1–10 (M,SD)	5.5 (2.9)	4.6 (2.7)	6.4 (2.7)
Education			
Low (%)	22.0	23.9	20.2
Middle (%)	48.0	44.8	51.2
High (%)	30.0	31.3	28.6

**Figure 2:**
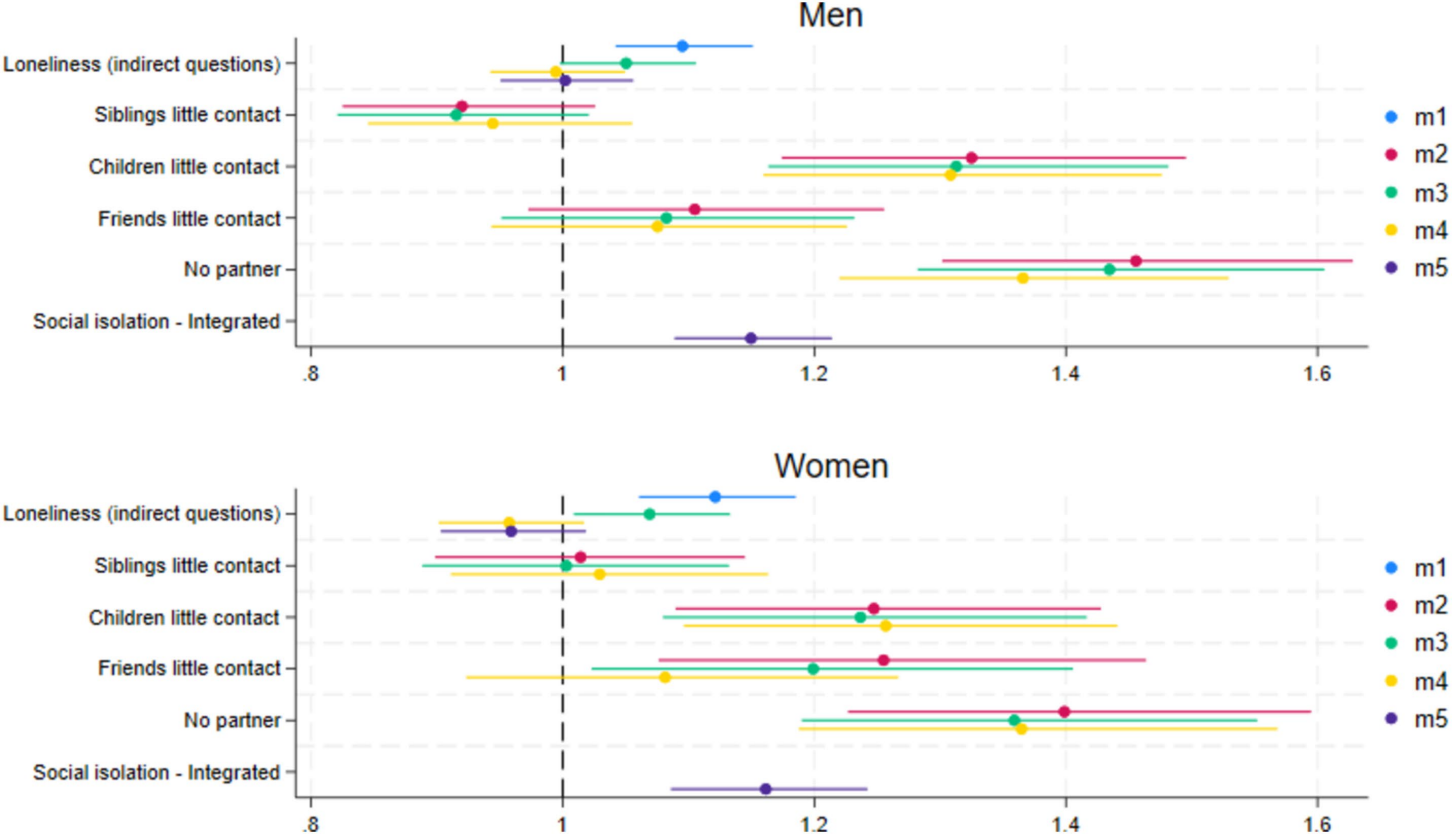
Coefficient plots of the hazard ratios for loneliness (indirect questions) and social isolation (index and disintegrated) for men and women. M1, Loneliness (+age); M2, Disintegrated social isolation (+age); M3, Loneliness and disintegrated social isolation (+age); M4, Loneliness and disintegrated social isolation + controls; M5, Loneliness and social isolation index + control.

In the first series of gender stratified Cox-regressions (Table 2) we examined the mortality risks of loneliness measured with indirect questions, plus the four indicators of social isolation and the social isolation index. Physical and mental health, income and education were added in the last models. The point estimates and 95% confidence intervals (CI) of the indirect questions about loneliness and social isolation coefficients can be most readily seen in Figure 2. More detailed information is in the table (Table 2).

**Table 2:** Cox proportional hazard regression of loneliness (indirect questions) and social isolation (index and disintegrated) on 20-years mortality.

Women (*n* = 5,079)	M1			M2			M3			M4			M5			M6		
	Loneliness			Social isolation			=M1 + M2			=M3+ controls			=M1 + SI Index+controls			=M5+ Interaction		
	HR	95% C.I.	*p*	HR	95% C.I.	*p*	HR	95% C.I.	*p*	HR	95% C.I.	*p*	HR	95% C.I.	*p*	HR	95% C.I.	*p*
Loneliness indirect	1.12	1.06, 1.19	0.000				1.07	1.01, 1.13	0.024	0.96	0.90, 1.02	0.157	0.96	0.90, 1.02	0.172	0.92	0.82, 1.03	0.157
Siblings little contact				1.01	0.90, 1.14	0.819	1.00	0.89, 1.13	0.963	1.03	0.91, 1.16	0.641						
Children little contact				1.25	1.09, 1.43	0.001	1.24	1.08, 1.42	0.002	1.26	1.10, 1.44	0.001						
Friends little contact				1.26	1.08, 1.46	0.004	1.20	1.02, 1.41	0.025	1.08	0.92, 1.27	0.332						
No partner				1.40	1.23, 1.59	0.000	1.36	1.19, 1.55	0.000	1.36	1.19, 1.57	0.000						
SI-index													1.16	1.09, 1.24	0.000	1.10	0.96, 1.27	0.154
Loneliness indirect * SI-Index																1.02	0.97, 1.08	0.406
Age	1.12	1.11, 1.13	0.000	1.12	1.11, 1.12	0.000	1.12	1.11, 1.12	0.000	1.11	1.10, 1.12	0.000	1.11	1.10, 1.12	0.000	1.11	1.10, 1.12	0.000
MCS12										0.98	0.97, 0.99	0.000	0.98	0.97, 0.98	0.000	0.98	0.97, 0.98	0.000
PCS12										0.97	0.97, 0.98	0.000	0.98	0.97, 0.98	0.000	0.98	0.97, 0.98	0.000
Income decile Education (Ref: Middle)										1.00	0.97, 1.03	0.937	1.01	0.98, 1.05	0.375	1.01	0.98, 1.05	0.357
Low										1.14	1.00, 1.31	0.047	1.17	1.03, 1.34	0.019	1.17	1.03, 1.34	0.019
High										0.74	0.60, 0.89	0.002	0.72	0.59, 0.87	0.001	0.72	0.59, 0.87	0.001
Number of observations	79, 033			79, 033			79, 033			79, 033			79, 033			79, 033		
Individuals	5, 079			5, 079			5, 079			5, 079			5, 079			5, 079		
AIC	15147.85			15125.61			15123.10			14963.66			14969.70			14971.09		
BIC	15166.40			15172.00			15178.77			15065.71			15043.92			15054.59		
Log pseudolikelihood	−7571.92			−7557.81			−7555.55			−7470.83			−7476.85			−7476.55		

Men (*n* = 4,872)	M1			M2			M3			M4			M5			M6		
	Loneliness			Social isolation			=M1 + M2			=M3+ controls			=M1 + SI Index+controls			=M5+ Interaction		
	HR	95% C.I.	*p*	HR	95% C.I.	*p*	HR	95% C.I.	*p*	HR	95% C.I.	*p*	HR	95% C.I.	*p*	HR	95% C.I.	*p*
Loneliness indirect	1.10	1.04, 1.15	0.000				1.05	1.00, 1.11	0.061	0.99	0.94, 1.05	0.841	1.00	0.95, 1.06	0.942	0.92	0.84, 1.01	0.086
Siblings little contact				0.92	0.82, 1.03	0.133	0.92	0.82, 1.02	0.112	0.94	0.85, 1.06	0.312						
Children little contact				1.33	1.17, 1.50	0.000	1.31	1.16, 1.48	0.000	1.31	1.16, 1.48	0.000						
Friends little contact				1.11	0.97, 1.26	0.125	1.08	0.95, 1.23	0.230	1.08	0.94, 1.23	0.277						
No partner				1.46	1.30, 1.63	0.000	1.43	1.28, 1.61	0.000	1.37	1.22, 1.53	0.000						
SI-index													1.15	1.09, 1.21	0.000	1.02	0.91, 1.16	0.695
Loneliness indirect * SI-Index																1.05	1.00, 1.11	0.042
Age	1.12	1.11, 1.13	0.000	1.12	1.11, 1.13	0.000	1.12	1.11, 1.13	0.000	1.11	1.11, 1.12	0.000	1.11	1.11, 1.12	0.000	1.11	1.11, 1.12	0.000
MCS12										0.99	0.98, 1.00	0.002	0.99	0.98, 0.99	0.001	0.99	0.98, 1.00	0.001
PCS12										0.97	0.97, 0.98	0.000	0.97	0.97, 0.98	0.000	0.97	0.97, 0.98	0.000
Income decile Education (Ref: Middle)										0.96	0.94, 0.99	0.004	0.96	0.94, 0.98	0.001	0.96	0.94, 0.98	0.001
Low										1.09	0.96, 1.24	0.199	1.11	0.97, 1.26	0.125	1.11	0.97, 1.26	0.123
High										0.81	0.70, 0.94	0.005	0.79	0.68, 0.91	0.001	0.79	0.68, 0.91	0.001
Number of observations	70,917			70,917			70,917			70,917			70,917			70,917		
Individuals	4,872			4,872			4,872			4,872			4,872			4,872		
AIC	19,380			19,315			19,314			19,114			19,145			19,142		
BIC	19,398.13			19,361.30			19,369.30			19,215.16			19,217.92			19,224.99		
Log pseudolikelihood	−9,687.90			−9,652.73			−9,651.14			−9,546.15			−9,564.28			−9,562.23		

HR, hazard ratio; C.I., Confidence interval; *p*, *p*-value; SI-Index, Social Isolation Integrated; MCS12, mental health; PCS12, Physical health; AIC, Akaike information criterion; BIC, Bayes information criterion.

The first model (M1, Table 2) shows that for each increase on the loneliness scale, the unconditional mortality risk of loneliness (indirect questions) becomes 12% higher for women (HR = 1.12, 95% CI 1.06–1.19) and 10% higher for men (HR = 1.10, 95% CI = 1.04–1.15). These effects remained significant after controlling for social isolation (M3). However, for men the effect is only borderline significant (HR = 1.05, 95% CI 1.00–1.11). In the second model (M2), the unconditional mortality risk of social isolation for women was statistically significant for having little contact with children (HR = 1.25, 95% CI = 1.09–1.43), friends (HR = 1.26, 95% CI = 1.08–1.46) and not having a partner (HR = 1.40, 95% CI = 1.23–1.59). The unconditional mortality risk of social isolation for men was statistically significant for having little contact with children (HR = 1.33, 95% CI = 1.17–1.50) and not having a partner (HR = 1.46, 95% CI = 1.30–1.63). The effects of social isolation on mortality remained statistically significant when controlling for loneliness in the third model (M3). When additionally controlling for mental and physical health, income, and education (M4), the effect of loneliness on mortality was no longer significant for both genders. For social isolation in M4, both little contact with children and not having a partner remained statistically significant predictors for mortality for both men and women. In the fifth model (M5), including the social isolation index instead of the four indicators of social isolation and loneliness (indirect questions), social isolation, but not loneliness (indirect questions) significantly increases the risk of mortality in both men and women while controlling for age, mental and physical health, income, and education.

**Figure 3:**
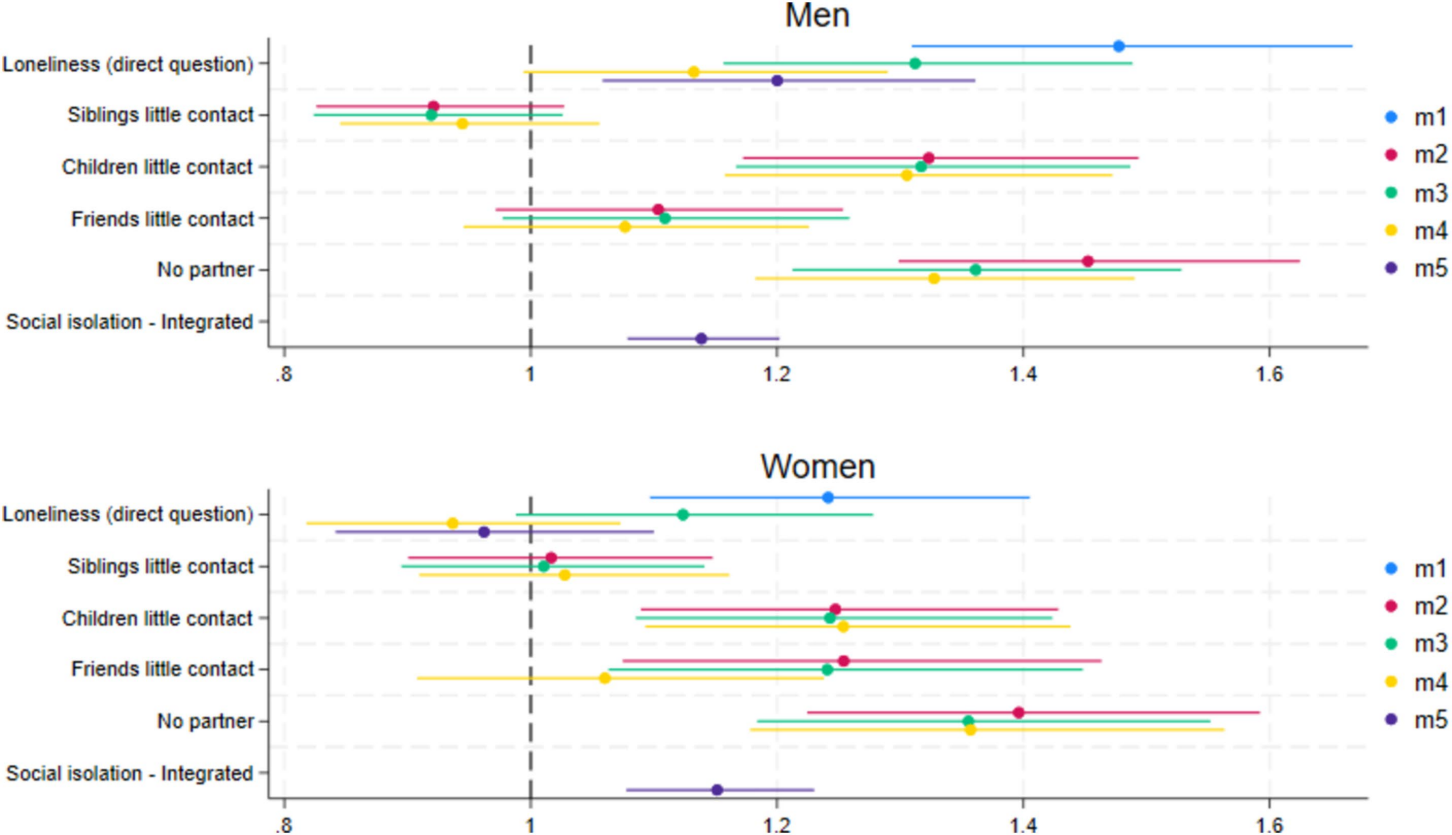
Coefficient plots of the hazard ratios for loneliness (direct question) and social isolation (index and disintegrated) for men and women. M1, Loneliness (+age); M2, Disintegrated social isolation (+age); M3, Loneliness and disintegrated social isolation (+age); M4, Loneliness and disintegrated social isolation + controls; M5, Loneliness and social isolation index + controls.

When repeating the same steps, but with loneliness assessed with a direct question, we find roughly the same pattern of associations with mortality for women (Figure 3 and Table 3). The statistically significant unconditional mortality risk of loneliness becomes insignificant when controlling for social isolation and all covariates (M4). The risk of not having a partner and little contact with children and friends (M3) remains significant when controlling for loneliness but having little contact with friends loses significance when controlling for all covariates (M4). For men, however, we find that when loneliness is measured with a direct question, loneliness remains a significant risk factor for mortality in the fully controlled model (M5; HR 1.20, 95% CI = 1.06–1.36), or borderline significant in M4 with the disintegrated social isolation index (HR = 1.13, 95% CI = 0.99–1.29).

**Table 3:** Cox proportional hazard regression of loneliness (direct question) and social isolation (index and disintegrated) on 20-years mortality.

Women (*n* = 5,076)	M1			M2			M3			M4			M5			M6		
	Loneliness			Social isolation			=M1 + M2			=M3+ controls			=M1 + SI Index+controls			=M5+ Interaction		
	HR	95% C.I.	*p*	HR	95% C.I.	*p*	HR	95% C.I.	*p*	HR	95% C.I.	*p*	HR	95% C.I.	*p*	HR	95% C.I.	*p*
Loneliness (direct)	1.24	1.10, 1.41	0.001				1.12	0.99, 1.28	0.076	0.94	0.82, 1.07	0.345	0.96	0.84, 1.10	0.572	1.08	0.83, 1.40	0.564
Siblings little contact				1.02	0.90, 1.15	0.790	1.01	0.89, 1.14	0.866	1.03	0.91, 1.16	0.661						
Children little contact				1.25	1.09, 1.43	0.001	1.24	1.09, 1.42	0.002	1.25	1.09, 1.44	0.001						
Friends little contact				1.25	1.07, 1.46	0.004	1.24	1.06, 1.45	0.006	1.06	0.91, 1.24	0.460						
No partner				1.40	1.22, 1.59	0.000	1.36	1.18, 1.55	0.000	1.36	1.18, 1.56	0.000						
SI-index													1.15	1.08, 1.23	0.000	1.18	1.09, 1.28	0.000
Loneliness direct * SI-Index																0.94	0.82, 1.07	0.319
Age	1.12	1.11, 1.13	0.000	1.12	1.11, 1.12	0.000	1.12	1.11, 1.12	0.000	1.11	1.10, 1.12	0.000	1.11	1.10, 1.12	0.000	1.11	1.10, 1.12	0.000
MCS12										0.98	0.97, 0.99	0.000	0.98	0.97, 0.99	0.000	0.98	0.97, 0.99	0.000
PCS12										0.98	0.97, 0.98	0.000	0.98	0.97, 0.98	0.000	0.98	0.97, 0.98	0.000
Income decile Education (Ref: Middle)										1.00	0.97, 1.03	0.956	1.01	0.98, 1.05	0.361	1.01	0.98, 1.05	0.378
Low										1.14	1.00, 1.30	0.054	1.17	1.02, 1.33	0.023	1.17	1.02, 1.33	0.023
High										0.73	0.60, 0.89	0.002	0.72	0.59, 0.87	0.001	0.72	0.59, 0.87	0.001
Number of observations	78, 976			78, 976			78, 976			78, 976			78, 976			78, 976		
Individuals	5, 076			5, 076			5, 076			5, 076			5, 076			5, 076		
AIC	15137.14			15111.26			15110.37			14950.13			14956.33			14957.43		
BIC	15155.69			15157.64			15166.04			15052.18			15030.55			15040.92		
Log pseudolikelihood	−7566.57			−7550.63			−7549.19			−7464.07			−7470.17			−7469.71		

Men (*n* = 4,872)	M1			M2			M3			M4			M5			M6		
	Loneliness			Social isolation			=M1 + M2			=M3+ controls			=M1 + SI Index+controls			=M5+ Interaction		
	HR	95% C.I.	*p*	HR	95% C.I.	*p*	HR	95% C.I.	*p*	HR	95% C.I.	*p*	HR	95% C.I.	*p*	HR	95% C.I.	*p*
Loneliness (direct)	1.48	1.31, 1.67	0.000				1.31	1.16, 1.49	0.000	1.13	0.99, 1.29	0.061	1.20	1.06, 1.36	0.004	1.00	0.78, 1.28	0.998
Siblings little contact				0.92	0.83, 1.03	0.139	0.92	0.82, 1.03	0.133	0.94	0.85, 1.06	0.315						
Children little contact				1.32	1.17, 1.49	0.000	1.32	1.17, 1.49	0.000	1.31	1.16, 1.47	0.000						
Friends little contact				1.10	0.97, 1.25	0.129	1.11	0.98, 1.26	0.109	1.08	0.95, 1.23	0.265						
No partner				1.45	1.30, 1.62	0.000	1.36	1.21, 1.53	0.000	1.33	1.18, 1.49	0.000						
SI-index													1.14	1.08, 1.20	0.000	1.10	1.04, 1.18	0.002
Loneliness direct * SI-Index																1.12	0.99, 1.27	0.081
Age	1.12	1.11, 1.13	0.000	1.12	1.11, 1.13	0.000	1.12	1.11, 1.13	0.000	1.11	1.11, 1.12	0.000	1.11	1.11, 1.12	0.000	1.11	1.11, 1.12	0.000
MCS12										0.99	0.98, 1.00	0.015	0.99	0.98, 1.00	0.011	0.99	0.98, 1.00	0.013
PCS12										0.97	0.97, 0.98	0.000	0.97	0.97, 0.98	0.000	0.97	0.97, 0.98	0.000
Income decile Education (Ref: Middle)										0.96	0.94, 0.99	0.005	0.96	0.94, 0.98	0.001	0.96	0.94, 0.98	0.001
Low										1.09	0.96, 1.24	0.182	1.11	0.97, 1.26	0.121	1.10	0.97, 1.25	0.131
High										0.81	0.70, 0.94	0.005	0.79	0.69, 0.92	0.002	0.79	0.69, 0.92	0.002
Number of observations	70,894			70,894			70,894			70,894			70,894			70,894		
Individuals	4,871			4,871			4,871			4,871			4,871			4,871		
AIC	19334.96			19296.33			19281.90			19094.65			19120.59			19119.58		
BIC	19353.30			19342.18			19336.92			19195.51			19193.95			19202.10		
Log pseudolikelihood	−9665.48			−9643.17			−9634.95			−9536.33			−9552.30			−9550.79		

HR, hazard ratio; C.I., Confidence interval; *p*, *p*-value; SI-Index, Social Isolation Integrated; MCS12, mental health; PCS12, Physical health; AIC, Akaike information criterion; BIC, Bayes information criterion.

In the last step of the Cox-regressions (M6) we evaluated whether an interaction between social isolation and loneliness would further increase mortality risks beyond the individual effects of loneliness and social isolation. All other factors but none of the interactions were significant accept one borderline significant interaction for men (M6 Tabel 2) (HR = 1.05, 95% CI = 1.00–1.11).

Finally, a robustness check was conducted to evaluate the impact of our handling of missing values in the Cox regressions, without carrying forward the last observation to the next round if respondents did not respond to a follow-up round without having died. This sample included 5,076 women and 4,870 men, of which 561 women and 814 men died during follow-up. While there were changes in the estimated confidence intervals, the point-estimates were rather similar and consistent with our conclusions.

## Discussion

With this study we examined the unique and synergistic effects of loneliness and social isolation on mortality in older men and women. We used and compared the effects of direct and indirect assessments of loneliness on mortality, as well as those between an index of social isolation and the disaggregated items and controlled for several well-known risk factors of mortality. For that purpose, we used a large population-based sample with 20-year follow-up information on mortality in older men and women.

During the 20 years of follow up, 1,008 (19.8%) women and 1,295 (26.6%) men deceased, which is in line with the well-known longer life expectancy for women. For women, we found that those who were socially isolated had approximately 15% higher mortality risk in the 20 years of follow up, irrespective of age, loneliness, mental and physical health, income, and education. The effect of social isolation on mortality was mainly driven by the absence of a partner and no or less than monthly contact with own children in the fully controlled models. While loneliness was initially associated with a higher mortality risk for women, even after controlling for social isolation, the effect of loneliness measured with direct and indirect questions was then fully explained by the other pertinent risk factors of mortality included in the analytical models. There was no evidence for a synergistic effect between loneliness and the social isolation as the interaction effects between the indirect loneliness scale and the social isolation index were insignificant.

For men we observed, similar to women, that the mortality risk of social isolation was around 15%, which was mainly driven by the absence of a partner, and less often than monthly contact with children. As for women, the initial mortality risk of loneliness measured with indirect questions for men became insignificant in the fully controlled models, and there was no evidence for a synergistic effect between indirect loneliness measures and social isolation. However, when loneliness was measured with the direct question a gender difference appeared. While for women, the impact of loneliness on mortality was fully explained by social isolation, education, mental and physical health, and income, for men the direct loneliness question remained a significant predictor of mortality in the fully controlled model. Not many studies examined gender differences in mortality risks of loneliness and social isolation that could help to interpret these differences. As far as we are aware, the two other studies examining gender differences in mortality risks of loneliness and social isolation (9, 10) did not find gender differences, which may be due to shorter follow-up (respectively 5 and 6.5 years) in these studies.

The differential impact of loneliness and social isolation on mortality suggests that the pathways from loneliness and social isolation to mortality are unique. The link between loneliness and mortality runs at least partly through mental health, to which loneliness is closely related, as loneliness loses significance when mental health and other control variables are added to the model. For social isolation, the impact on mortality remains significant, even after controlling for all other variables, suggesting a unique effect of social isolation beyond possible impacts of loneliness and other related factors. An important difference between loneliness and social isolation is that lonely people may still be surrounded by other people, despite the perceived lack of quality or quantity of social relations. Socially isolated people are merely on their own. And whether they are at peace with their social situation or not, they are deprived of support and people who can act in acute life-threatening conditions such as with falls, strokes, or heart attacks. As we do not know the precise cause of death, this assumption could not be tested in the present study. We found little support for the suggestion by Newall and Menec (23) that people who are both isolated and lonely are the most vulnerable, and therefore may have the highest mortality risk. The synergistic effects were not even close to significant for women, and only borderline significant for men if loneliness is measured with indirect questions.

Another important finding of our study was that loneliness has a unique effect on mortality for men only when measured with a direct question that is including the word lonely, but not with questions avoiding the word lonely. A straightforward explanation is that the difference in effect lies in the formulation of the questions. People, especially men (31) may not like to admit that they are lonely because of the social taboo that still rests on loneliness (32, 33). But if people openly admit that they are lonely, loneliness may be severe with significant impacts on mortality. Two studies evaluating the severity of loneliness indeed find that loneliness only increases mortality if it is chronic or severe (34, 35).

An alternative explanation is that the two loneliness measures reflect distinct facets of loneliness with varying associations with mortality. To sort this out, a critical appraisal and validation of existing measurement instruments of loneliness and social isolation is needed. Some good initiatives in this field can be found in the studies by Maes et al. (36), and Mund et al. (22). They conclude that while direct and indirect questions about loneliness can provide valid estimates of loneliness, it is not always clear which type of loneliness is measured. A difference in power or measurement error between the two measures of loneliness seems an unlikely explanation, as it is precisely the less-sensitive single item question with the smallest variance and probably the largest measurement error.

The inclusion of separate indicators for social isolation allowed us to define which aspect of social isolation is most relevant for mortality for older women and men. Our models confirm the long-established risk of not having a partner on mortality. For both women and men in our study, not having a partner was the most important risk factor for mortality and increased the risk of dying with around 40% (women) and 45% (men). Explanations that can be found in the literature for the protective effect of the partner range from the provision of financial and social support, stress of bereavement or divorce, to stimulation of healthier lifestyles [e.g. (37)]. Men had a 33% higher mortality risk if there was only little or no contact with children and women had 25% higher mortality risk if this was the case. Children are important resources for support in later life, and a low contact frequency may therefore lead to support deficits, which in turn might increase mortality. However, children are not the only resources for support and a support deficit from children may be compensated with support from other people from the network, such as friends. There is evidence that women get support from several close network members whereas men often rely on the closest person (partner or children) (38). Indeed, in our unconditional model, having frequent contact with friends was associated with lower mortality risks for women, but not for men.

Our study had limitations which are important to consider when interpreting the results. While our study sheds some light on the inconsistencies in previous studies on the lethal impact of loneliness and social isolation, it did not solve all issues. For example, in the meta-analysis by Wang et al. (7), 16 of the 90 included studies reported a significant mortality risk of loneliness, but only half of these studies used direct questions including the word lonely. The use of direct or indirect measures of loneliness is thus an important but not sufficient reason for the discrepancies. An alternative explanation is that relevant “third variables” or residual confounding was not considered which could have contributed to different results. While we controlled for several individual-level third variables and stratified by gender, macro-level factors may further explain differences in findings between national studies. For example, studies on loneliness consistently find that loneliness is highest in countries were living alone is most prevalent [e.g., (39–44)], suggesting a differential impact of living alone on loneliness in different countries. Interpretations of these findings vary from structural differences (e.g., welfare state provisions, living standards) to cultural (e.g., familistic vs. individualistic cultures, differences in interpersonal trust). We could not take these macro factors into account as we have only data from one country (Norway). Finally, our indirect measurement of loneliness was based on three of the original 11 items of the De Jong Gierveld loneliness scale (45). We are not aware of validation studies confirming that loneliness was indeed latent construct influencing the scores of these three items.

To conclude, our study confirms that social isolation leads to higher mortality in both men and women, controlling for loneliness and other well-known risk factors, and this effect is mainly driven by not having a partner. Little contact with children further contributes to higher mortality. If loneliness is assessed with indirect questions, that is with questions not including the word “lonely,” loneliness loses its predictive significance when other well-known risk factors are considered in tandem. When measured with a direct question, the mortality risk of loneliness remains only significant for men in the fully controlled models. The results of our study suggests that selecting indicators for social isolation should be done with care, as not all indicators are relevant for mortality and gender differences exist in the impact of different aspects of social isolation. Moreover, our findings indicate that direct and indirect questions about loneliness might tap into different aspects of loneliness, or into different degrees of severity, at least for men. The results highlight the critical importance of methodological precision in measuring loneliness and social isolation. While many surveys include the direct question of loneliness for practical reasons, researchers as well as policy makers and care practitioners using the direct question should be aware that outcomes may be different when indirect questions or different indicators for social isolation about loneliness are used.

## Data Availability

Publicly available datasets were analyzed in this study. This data can be found here: https://norlag.nsd.no/ signing a data distribution contract is required. The dataset is available for research purposes from the Norwegian Agency for Shared Services in Education and Research.
